# Metabolomics Study of Different Germplasm Resources for Three *Polygonatum* Species Using UPLC-Q-TOF-MS/MS

**DOI:** 10.3389/fpls.2022.826902

**Published:** 2022-03-11

**Authors:** Shiqiang Wang, Wenna Li, Xinfei Zhang, Gang Li, Xiao dong Li, Hui Chang, Junfeng Niu, Zhezhi Wang

**Affiliations:** ^1^National Engineering Laboratory for Resource Development of Endangered Crude Drugs in Northwest China, The Key Laboratory of Medicinal Resources and Natural Pharmaceutical Chemistry, The Ministry of Education, College of Life Sciences, Shaanxi Normal University, Xi’an, China; ^2^Lueyang Chinese Herbal Medicine Industry Development Service Center, Hanzhong, China; ^3^Shaanxi Buchang Pharmaceuticals Limited Company, Xi’an, China

**Keywords:** *Polygonatum*, species discrimination, metabolomics, UPLC-Q-TOF-MS/MS, iconic metabolites

## Abstract

Rhizomes of the *Polygonatum* species are well-known in traditional Chinese medicine. The 2020 edition of *Chinese Pharmacopoeia* includes three different species that possess different pharmacological effects. Due to the lack of standardized discriminant compounds there has often been inadvertently incorrect prescriptions given for these medicines, resulting in serious consequences. Therefore, it is critical to accurately distinguish these herbal *Polygonatum* species. For this study, UPLC-Q-TOF-MS/MS based metabolomics was employed for the first time to discriminate between three *Polygonatum* species. Partial least squares discriminant analysis (PLS-DA) models were utilized to select the potential candidate discriminant compounds, after which MS/MS fragmentation patterns were used to identify them. Meanwhile, metabolic correlations were identified using the R language package corrplot, and the distribution of various metabolites was analyzed by box plot and the Z-score graph. As a result, we found that adenosine, sucrose, and pyroglutamic acid were suitable for the identification of different *Polygonatum* species. In conclusion, this study articulates how various herbal *Polygonatum* species might be more accurately and efficiently distinguished.

## Introduction

Rhizomes of the *Polygonatum* species are well-known traditional Chinese medicines that have been extensively applied for the treatment of many diseases over hundreds of years in China, Korea, Japan, and other East Asian countries (Zhao and Li, 2015). Approximately, 60 species have been identified worldwide, 31 of which are found in China. However, only three species have been selected for *Chinese Pharmacopoeia*. These include *Polygonatum sibiricum* Delar. ex Redoute, *Polygonatum cyrtonema* Hua, and *Polygonatum kingianum* Coll. et Hemsl.

The herb Huangjing. *P. sibiricum* can assist with promoting the proliferation and enhancing the viability of Bone Mesenchymal Stem Cells (BMSCs) (Zong et al., 2015), prevent Alzheimer’s disease (Zhang et al., 2015), osteoporosis (Zeng et al., 2011), hypolipidemia and atherosclerosis (Yang et al., 2015), and improve immunologic functions (Wu et al., 2014). *P. cyrtonema* can induce apoptosis and autophagy in human lung adenocarcinoma A549 cells (Liu et al., 2016), whereas *P. kingianum* may be applied to prevent T2D by its regulatory role in gut microbiota (Yan et al., 2017).

Furthermore, other *Polygonatum* species have a history of medicinal use, with various species having different medicinal effects. *P. cirrhifolium* can reduce oxidative stress for the protection of neurons (Xu et al., 2017); *P. odoratum* can be used to inhibit the proliferation of cancer cells (Tai et al., 2016); and *P. stenophyllum* can suppress menopausal obesity (Lee et al., 2016).

Since standardized discriminant compounds are not yet available for the *Polygonatum* species, they are distinguished only by their physiological appearance and morphological features. However, due to the very similar physical features and distribution areas of some *Polygonatum* species, this has often led to prescription confusion, with serious consequences (Zhao et al., 2006). Although some researchers have focused on elucidating the discriminant compounds of *Polygonatum*, they have not yet been successfully employed to accurately differentiate *Polygonatum* species. Thus, it is imperative to distinguish between closely related herbal medicines. Currently, most studies on *Polygonatum* have focused on the isolation of active components and their functions. According to the *Chinese Pharmacopoeia*, polysaccharide is the main medicinal component of *Polygonatum*, so we measured the polysaccharide content of different *Polygonatum*. However, the determination of polysaccharide content alone could not distinguish the different *Polygonatum* species. So we want to find out discriminant compounds to distinguish *Polygonatum* species more accurately. With the development of biometric techniques, the identification of medicinal plants based on their signature components is becoming increasingly popular. Ultra-performance liquid chromatography/quadrupole time-of-flight mass spectrometry (UPLC/Q-TOF MS) offers higher resolution and sensitivity; thus, UPLC-Q-TOF-MS/MSbased metabolomics has been used to determinate the raw materials of various plants, including *Salvia officinalis* (Sarrou et al., 2017), *Cimicifugae Rhizoma* (Fan et al., 2017), *Carthamus tinctorius* (Yao et al., 2017), and *Plantago* (Yao et al., 2017).

For this study, UPLC-Q-TOF-MS/MS-based metabolomics was employed for the first time to accurately distinguish between herbal *Polygonatum* species (we chose the five *Polygonatum* with the significant difference of polysaccharide content, three of them are the same species but different regions), with analytical results revealing the most suitable metabolites that could reliably discriminate between them.

## Materials and Methods

### Chemicals and Herbal Medicines

HPLC grade methanol was obtained from Merck (Germany), HPLC grade chloroform, formic acid, and acetonitrile were purchased from Thermo Fisher Scientific (United States), and ultrapure water generated by a Milli-Q Progard TS2 system (France) was used in all experiments.

Seventeen germplasm sources of *Polygonatum* were collected across China in 2015 (Supplementary Table 1), which were authenticated by Prof. Yaping Xiao (Key Laboratory of the Ministry of Education for Medicinal Resources and Natural Pharmaceutical Chemistry, Shaanxi Normal University). Voucher specimens were deposited at the National Engineering Laboratory for Resource Development of Endangered Chinese Crude Drugs in Northwest China, Xi’an, China.

### Isolation and Detection of Polysaccharides

The rhizome of *Polygonatum* were dried at 60°C for 48 h and ground to a powder, 100 g of which were mixed with petroleum ether (300 mL) at 60°C for 12 h to remove the lipids, which were then extracted via distilled water at 80°C for 3 h at a 1:30 ratio (w/v) and repeated twice. The water extract was collected and concentrated using a rotary evaporator. After concentrating the liquid, 95% ethanol was added to a fourfold volume, which was maintained at 4°C overnight to precipitate the polysaccharide and then percolated. The proteinaceous sediment was removed by repeated freezing/thawing, dialyzing, and lyophilizing to obtain the crude polysaccharide. The content of the polysaccharide was then calculated for *Polygonatum* (PSP).

PSP (%) content = W/M × 100%.

W, weight of crude polysaccharide.

M, weight of dried *Polygonatum* rhizome powder.

### Sample Preparation

According to the PSP content and four key indexes including leaf width, impeller number, total pedicle length and total flower number were taken into consideration, and the specific proportion weighting method was adopted to select the *Polygonatum sibiricum* Delar. ex Redoute from Lueyang County (Hanzhong, Shaanxi Province. Longitude: 33°17′56″; latitude: 105°51′12″; elevation: 1192 m), Foping County (Hanzhong, Shaanxi Province. Longitude: 33°52′86″; latitude: 108°01′71″; elevation: 1494 m), Luanchuan County (Luoyang, Henan Province. Longitude: 34°39′41″; latitude: 112°24′7″; elevation: 1193 m), *Polygonatum cyrtonema* Hua from Meishan City (Sichuan Province. Longitude: 30°06′02″; latitude: 103°86′13″; elevation: 1278 m) and *Polygonatum kingianum* Coll. et Hemsl from Lincang (Yunnan Province. Longitude: 23°87′58″; latitude: 100°06′94″; elevation: 1527 m) as the metabolomic samples, they are abbreviated to PSLY, PS (PSFP), PSLC, PC, and PK, and repeated the processing of each sample six times. The rhizome samples were placed in liquid nitrogen and then ground into fine powder, after which 80 mg of each sample was placed into a 5 mL glass centrifuge tube. Subsequently, 1 mL of 100% precooled (−20°C) methanol was added and the mixture underwent vortex oscillation for 30 s, which was then placed into the ultrasound unit at room temperature for 30 min. Chloroform (750 μL) was then added with ddH_2_O (4°C) 800 μL and underwent vortex oscillation for 1 min and centrifugation for 10 min at 10,000 rpm. The upper methanol/water mixture layer (1,000 μL) was transferred to a new 1.5 ml centrifuge tube, wherein the sample was concentrated using a vacuum centrifuge.

### Liquid Chromatography-Mass Spectrometry Analysis

Liquid chromatography analysis was performed using a SHIMADZU LC-30A Ultra High-Performance Liquid Chromatography system (SHIMADZU, Japan), equipped with a C18 column (1.7 μm, 2.1 × 100 mm) at 40°C, with a 4 μL injection volume. The mobile phase consisted of 0.1% formic acid water (A) and acetonitrile (B), using 2% B at 0–0.5 min, 2–50% B at 0.5–9 min, 50–98% B at 9–12 min, 98% B at 12–13 min, 98-2% B at 13 – 14 min, and 2% B at 14–15 min. The sample was dissolved in the mobile phase. The next sample was collected after 1 min of equilibration in the column. The flow rate was 0.3 mL/min and the autosampler temperature was maintained at 4°C.

The MS analysis was performed using an ABSCIEXTripleTOF™5600 LC/MS/MS system (United States) with a Duospray source. The Duospray source parameters included (ion voltage = 4,000 V and 5,500 V in negative and positive mode, declustering potential voltage was 80 V, source temperature was 600°C, curtain gas was 35 psi, Gas1 (nebulizer gas) was 60psi; Gas2 (heater gas) was 65psi; and the mass analyzer was scanned over m/z 50–1000, and the scan mode was MS/MS, IDA [each cycle scan response to the highest 8 ion scatter secondary scan)]. The dynamic background was excluded, and the instrument was recalibrated every five samples.

### Data Pretreatment and Multivariate Statistical Analysis

The raw LC/MS data of the 30 test samples were processed using XCMS^1^ for peak deconvolution and alignment (Supplementary Table 2). The parameters of XCMS are set as follows: (a) xset = xcmsSet [snthresh = 6, method = “centWave,” ppm = 15, mzdiff = 0.01, peakwidth = c(10,120)]; (b) group [xset, bw = 5, minfrac = 0.5, mzwid = 0.15]; (c) retcor [xset1,method = “obiwarp,” plottype = c (“deviation”)]; (d) group (xset2, bw = 5, minfrac = 0.5, mzwid = 0.15). 11,442 variables (positive ion mode) and 16,637 variables (negative ion mode) were obtained. The aligned data were exported to an Excel table and the peak area was normalized by the sum^2^. Principal component analysis (PCA) and partial least squares discriminant analysis (PLS-DA) were performed using SIMCA-P version 13.0 (Umetrics, Sweden) after Pareto scaling. Only variables with VIP > 1.0 and *P* < 0.05 were selected as discriminant compounds. Next, the MS/MS fragmentation patterns were employed as potential discriminant compounds in the Human Metabolome Database (HMDB)^3^ (Wishart et al., 2009), Metlin^4^ (Smith et al., 2005), and massbank^5^ (Horai et al., 2010) and LipidMaps^6^. The R language^7^ package Pheatmap was used to classify the identified potential discriminant compounds, after which the metabolic correlations were analyzed using the R language (see text footnote 7) package corrplot. For an intuitive view of the distribution of the different metabolites between groups, the box plot and the Z-score were used for analysis. Z-score is based on the relative conversion of metabolites and used to measure the relative level of metabolites. Z = (x – μ)/σ. Where x is a specific fraction, μ is the mean, and σ is the standard deviation.

## Results

### *Polygonatum* Content of Different Germplasms

In this study, the crude water-soluble polysaccharide was obtained from the dried rhizomes of different germplasms. The specific location data is depicted in Supplementary Table 1. As shown (Table 1), the PSP content of PSLY was highest (13.33%), while the PSLC was lowest (6.37%). The PSP content of the PC was 10.83%, while for the PK was 11.76%. All differences in the data above were significant. In order to better evaluate the differences in metabolites of the three *Polygonatum* species, according to the yield of polysaccharides and other biological traits, we selected the germplasms of PSLY, PSLC, PS, PC, and PK as the metabolomics samples, among them, PSLY, PSLC, and PS are the same species but different regions.

**TABLE 1:** Polysaccharide content of *P. sibiricum* in different germplasm samples.

No	Origin	Species	Abbreviation	PSP Content %
1	Xiakou Yi, ShaanXi, China	*P. sibiricum*	PSXKY	11.91 ± 0.13 deCD
2	Shangluo, ShaanXi, China	*P. sibiricum*	PSSL	11.63 ± 0.12 eDE
3	ZhenAn, ShaanXi, China	*P. sibiricum*	PSZA	12.11 ± 0.11 cdCD
4	Danfeng, ShaanXi, China	*P. sibiricum*	PSDF	11.2 ± 0.36 fEF
5	Liuba, ShaanXi, China	*P. sibiricum*	PSLB	8.85 ± 0.14 jI
6	Lueyang, ShaanXi, China	*P. sibiricum*	PSLY	13.33 ± 0.1 aA
7	Foping, ShaanXi, China	*P. sibiricum*	PS	12.65 ± 0.12 bB
8	Luoyang, HeNan, China	*P. sibiricum*	PSLC	6.37 ± 0.11 kJ
9	Anyang, HeNan, China	*P. sibiricum*	PSAY	10.39 ± 0.09 hG
10	Yingshan, HuBei, China	*P. sibiricum*	PSYS	10.85 ± 0.08 fgFG
11	Shiyan, HuBei, China	*P. sibiricum*	PSSY	8.62 ± 0.1 jI
12	Tiantai, ZheJiang, China	*P. sibiricum*	PSTT	12.4 ± 0.08 bcBC
13	Jiangxian, ShanXi, China	*P. sibiricum*	PSJX	10.65 ± 0.1 ghFG
14	Meishan, Sichuan, China	*P. cyrtonema*	PC	10.83 ± 0.08 fgFG
15	Guiling, Guangxi, China	*P. cyrtonema*	PCGL	9.48 ± 0.09 iH
16	Lincang, Yunnan, China	*P. kingianum*	PK	11.76 ± 0.07 deD
17	Wenshan, Yunnan, China	*P. kingianum*	PKWS	11.11 ± 0.14 fEF

*PS, Polygonatum sibiricum; PC, Polygonatum cyrtonema Hua; PK, Polygonatum kingianum Coll. et Hemsl.*

### Identification of Compounds Using UHPLC-Q-TOF-MS

**FIGURE 1:**
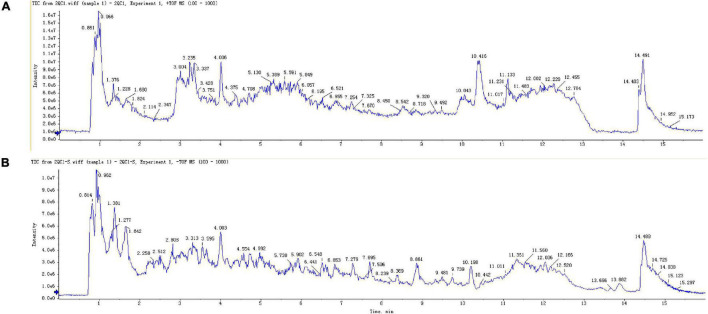
Total ions chromatogram of *P. sibiricum.* **(A)** Positive ions. **(B)** Negative ions.

An UHPLC-QTOF-MS technique was established and successfully applied to characterize the constituents of the methanol extract of rhizome of *Polygonatum*. Total ion flow chromatogram of *Polygonatum*, including positive and negative ion modes (Figure 1). As a result, 7,064 variables (positive ion mode) and 13,780 variables (negative ion mode) were obtained (Supplementary Table 2).

### Multivariate Statistical Analysis

**FIGURE 2:**
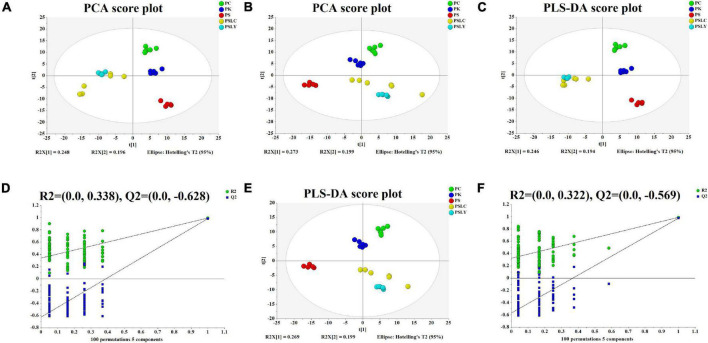
PCA and PLS-DA score plots and the PLS-DA permutation test chart. **(A)** Positive ion mode of PCA score plot (R^2^X = 0.735, Q^2^ = 0.572). **(B)** Negative ion mode of PCA score plot (R^2^X = 0.736, Q^2^ = 0.529). **(C)** Positive ion mode of PLS-DA score plot (R^2^X = 0.734, R^2^Y = 0.988, Q^2^ = 0.971). **(D)** Positive ion mode of PLS-DA permutation test. **(E)** Negative ion mode of PLS-DA score plot (R^2^X = 0.734, R^2^Y = 0.983, Q^2^ = 0.956). **(F)** Negative ion mode of PLS-DA permutation test.

**FIGURE 3:**
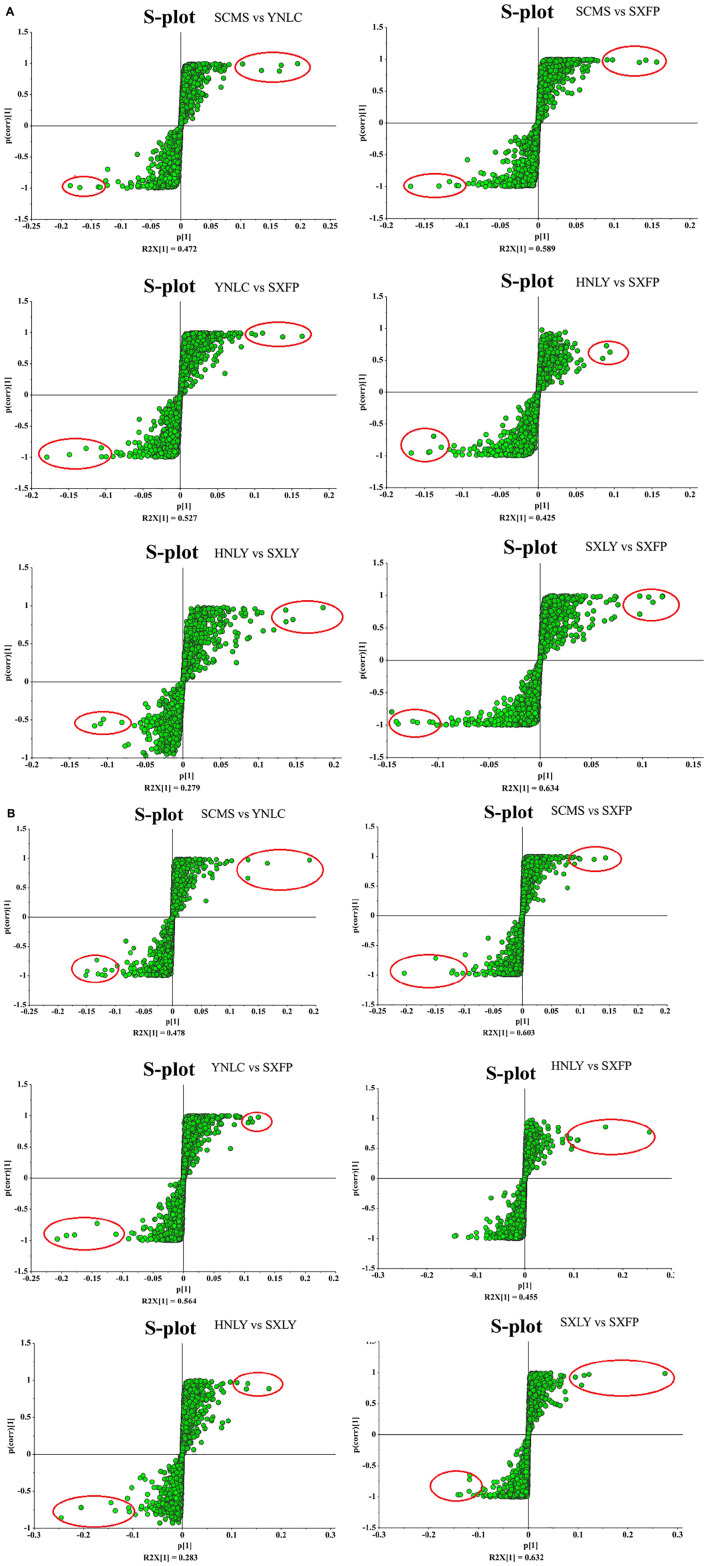
**(A)** S-plot analysis of different origins of *P. sibiricum* (positive ion mode). **(B)** S-plot analysis of different origins of *P. sibiricum* (negative ion mode).

**FIGURE 4:**
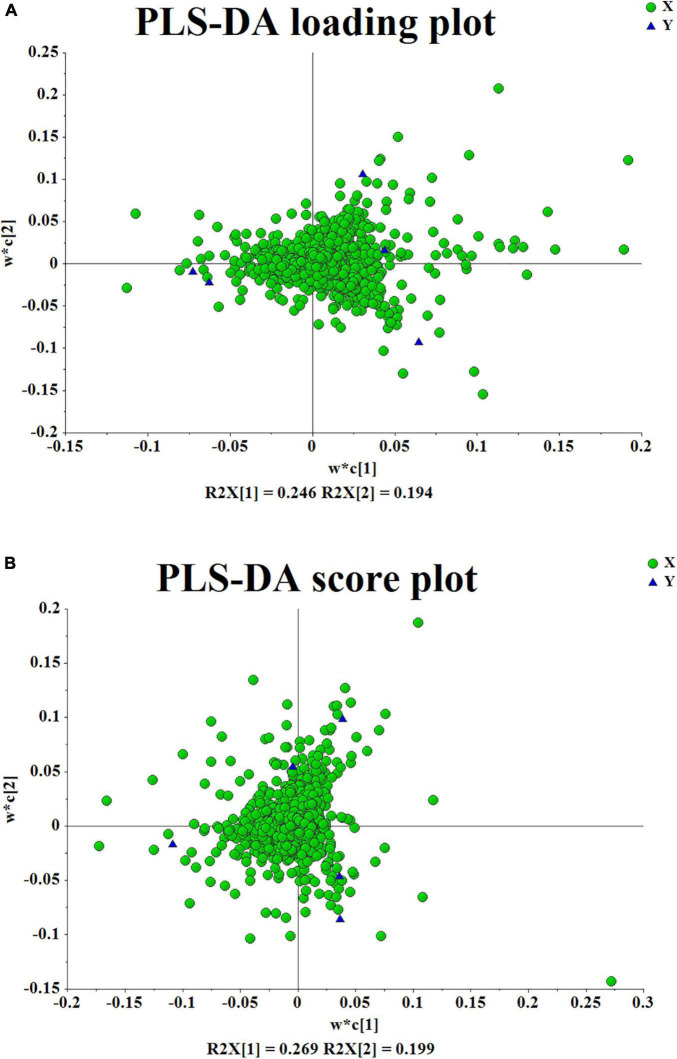
**(A,B)** The scattered loading plot.

After several pretreatment procedures the data were applied to PCA to visualize the grouping trends. In the automatic simulation process, positive ion obtained five principal components, R2X = 0.444, Q2 = 0.572; The negative ion obtained five principal components, R2X = 0.472, Q2 = 0.529. The samples are basically in the 95% confidence interval (Hotelling T^2^ Ellipse). As shown in Figures 2A,B, the PCA score plot showed a clear distinction between the PS, PC, and PK samples, while the separation between the PS, PSLY, and PSLC was not clear. To select the chemical markers responsible for such separation, the data set was applied to PLS-DA. PLS-DA for supervised analysis model of multivariate statistical analysis, compared with PCA can distinguish the differences between different metabolic category, image more clear can argue, and further to test the quality of the model using cross validation method, using the R2X (model can explain the variable) and Q2 (predictable degree) of the model for effectiveness evaluation model. R2X = 0.44, Q2 = 0.971 (positive ion mode), R2X = 0.468, Q2 = 0.956 (negative ion mode) (Figures 2C,E). The abscissa is the first principal component score, represented by PC1; The ordinate is the second principal component score, represented by PC2, and the parentheses are the percentage of the explained model. R2Y (interpretation rate of supervision model) indicates that PLS-DA model can well explain the differences between each group of samples. After that, permutation test was performed on the PLS-DA model, and 100 times of positive ion mode verification results showed that the intercept of R2 on the *Y*-axis was 0.338, and that of Q2 on the *Y*-axis was −0.628 (Figure 2D). The 100 times verification results of negative ion mode show that the intercept of R2 on the *Y*-axis is 0.322, and that of Q2 on the *Y*-axis is −0.569 (Figure 2F). R2X is the explanatory degree of model X variable, R2Y is the explanatory degree of model Y variable, and Q2 is the predictability of the model. Theoretically, the closer R2X and Q2 are to 1, the better the model will be. The significance of variables using PLS-DA first principal component (VIP > 1), and metabolites with significant differences were screened with *P* < 0.05. In Figures 3A,B, each square of s-plot represents one compound, and compounds closer to the lower left and upper right corner represent greater contributions to each group of classification. Finally, 43 potential biomarkers were screened out (Supplementary Table 3). The scattered loading plot is shown in Figure 4A (positive ion mode) and Figure 4B (negative ion mode).

### Structural Annotation and Quantification of Potential Chemical Markers

According to the MS/MS fragmentation models in the HMDB, Metlin, massbank, and LipidMaps databases, the potential biomarkers (Supplementary Table 3) were identified using the exact molecular weights. As shown in Supplementary Table 4 and Supplementary Figure 1, 25 key constituents were identified.

**FIGURE 5:**
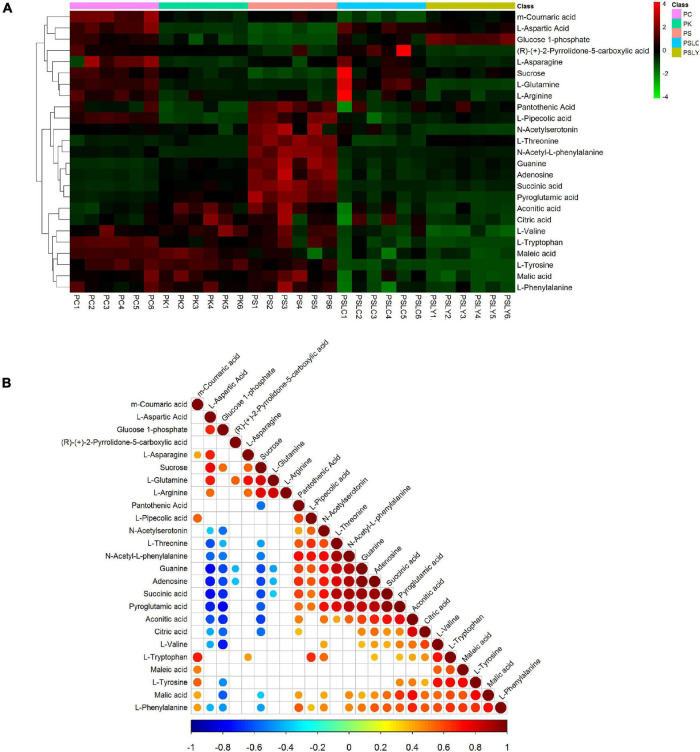
Heat map and correlation analysis chart of differential metabolite. **(A)** Differential metabolite heat map. **(B)** Correlation analysis chart.

As shown in Figure 5A, a heat map of the 25 key constituents was used to visualize the relative level trends for all test samples. The different colors represent the relative levels, with red hues being higher and green hues being lower. From Figure 5A, there were significant differences in the contents of the five types of *Polygonatum* (PS, PC, PK, PSLY, and PSLC). These included adenosine, citric acid, guanine, L-pipecolic acid, L-tryptophan, pyroglutamic acid, and sucrose. The screening results, comparing each *Polygonatum* group are shown in Table 2. The results revealed that the iconic differential metabolites suitable for the identification of different *Polygonatum* species were adenosine, sucrose, and pyroglutamic acid. The iconic differential metabolites that were suitable for the identification of *P. sibiricum* of different regions included sucrose, citric acid, and pyroglutamic acid. Further, adenosine, sucrose, and pyroglutamic acid were appropriate for all five types of *Polygonatum* to distinguish.

**TABLE 2:** Discrepant metabolites.

No.	mz	Rt (s)	exact_mass	mass_error	Metabolite	PC vs PK.vip	PC vs PS.vip	PK vs PS.vip	PSLC vs PS.vip	PSLC vs PSLY.vip	PSLY vs PS.vip
1	128.04	95.93	129.04	5	Pyroglutamic acid	5.20	7.71	7.06	8.75	4.07	8.80
2	130.09	83.12	129.08	1	L-Pipecolic acid	2.79	1.25	3.04	2.65	1.16	2.21
3	152.06	203.54	151.05	3	Guanine	1.39	2.41	2.43	2.54	1.04	2.18
4	191.02	85.06	192.03	3	Citric acid	8.17	4.90	1.14	4.05	5.26	7.36
5	205.10	253.37	204.09	3	L-Tryptophan	6.68	2.53	4.45	6.33	2.87	6.52
6	268.10	200.03	267.10	2	Adenosine	3.94	7.22	7.48	7.84	2.59	6.79
7	341.11	58.83	342.12	1	Sucrose	7.27	7.57	5.09	6.93	4.66	5.86

For the 25 key constituents, we calculated the Pearson correlation coefficient and statistic test P value using the R language software (see text footnote 7) package corrplot. The highest correlation coefficient was −1 (red), which represented a completely positive correlation, and the lowest correlation coefficient was −1 (blue), which represented a completely negative correlation. The blank portion of the figure is the correlation statistic test p value > 0.05, whereas the colored portion is *p* < 0.05.

As shown in Figure 5B, the correlation analysis of the 25 key constituents intuitively showed the association between the different metabolites. Here, we focused on the iconic differential metabolites that were suitable for identifying all five *Polygonatum* species. These included adenosine, sucrose, and pyroglutamic acid, where sucrose was negatively correlated with adenosine and pyroglutamic acid.

**FIGURE 6:**
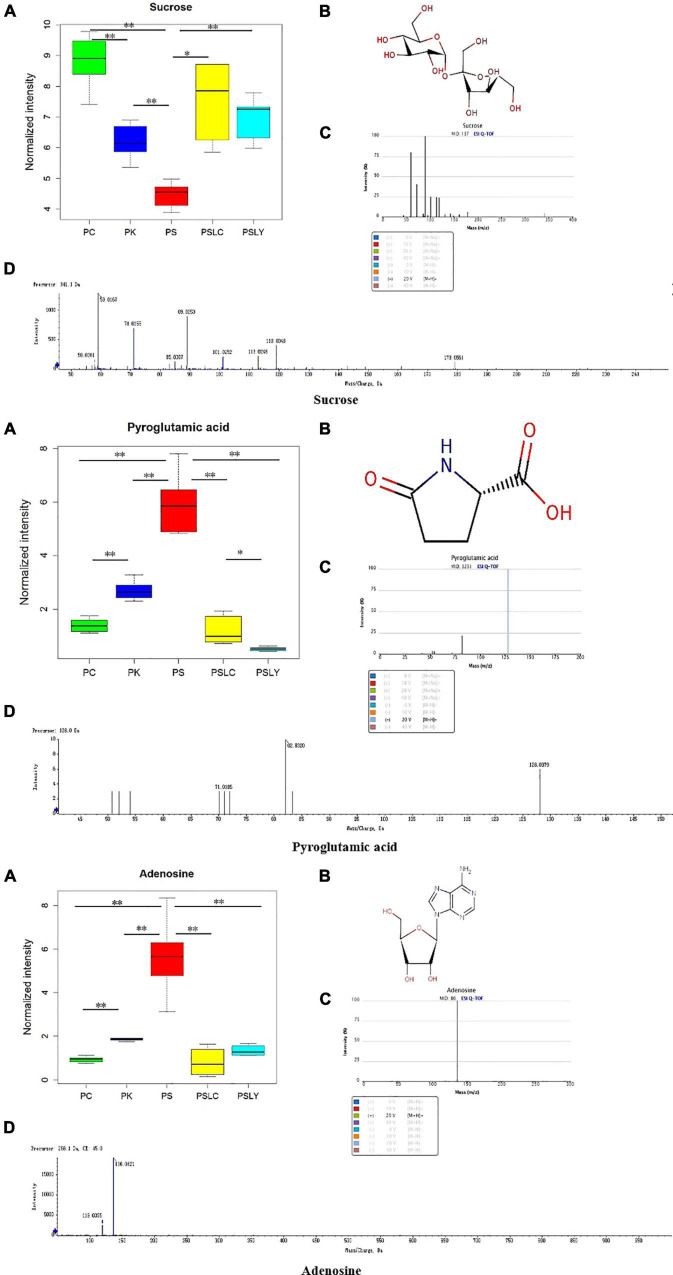
Box diagram, spectra, and structural formulas of novel markers. **(A)** Box diagram. **(B)** Structural formulas. **(C)** Standard spectra of novel markers. **(D)** Detection spectra of novel markers. *represents significant differences, **represents extremely significant differences.

For an intuitive view of the distribution of the different metabolites between groups, the box plot (Figure 6) was used for analysis. The box plot in Figure 6A shows the relative level of the three compounds in the *Polygonatum* extracts. The relative level of sucrose was highest in the *P. cyrtonema* extracts and about twofold higher than the *P. sibiricum* extracts, which had the lowest level between all *Polygonatum* species. However, the relative level of pyroglutamic acid and adenosine were highest in the *P. sibiricum* extracts. Finally, it was evident that the relative level of adenosine, sucrose, and pyroglutamic acid were significantly different for the five types of *Polygonatum*, which indicated that they could be employed for the identification of different *Polygonatum* species.

## Discussion

The metabolites of identical plants can vary when grown in different environments, for different organs, different producing areas, or through various processing methods (Souza et al., 2019). Consequently, the quality of the medicinal components of the *Polygonatum* species (particularly in terms of their chemical compositions) is uneven due to the mixed germplasm, transregional introduction, and diverse planting and processing procedures (Skubel et al., 2018). Moreover, traditional classification methods such as character (Xu et al., 2018), microscopic (Boiteau et al., 2018), and optical identification (Ullah et al., 2017), cannot directly reflect the nuances in their chemical compositions.

Metabolomics is a new discipline for the qualitative and quantitative analyses of small molecule metabolites, which has the advantages of high sensitivity, a low detection limit, and small sample requirements, and is mostly used for the detection and analysis of plant signature components (Tian et al., 2014; Li et al., 2015; Puzanskiy et al., 2017). Therefore, we applied it for the identification of different types of Chinese medicinal *Polygonatum*, to facilitate the more rapid and accurate identification of Chinese medicinal ingredients. It is of great significance to analyze the metabolites of different germplasms. According to the *Chinese Pharmacopoeia*, polysaccharide is the main medicinal component of *Polygonatum*, so we measured the polysaccharide content of different *Polygonatum*. However, the determination of polysaccharide content alone could not distinguish the different *Polygonatum* species. Therefore, we combine the differences in polysaccharide content, as well four key indexes including leaf width, impeller number, total pedicle length and total flower number were taken into consideration, and the specific proportion weighting method was adopted to select. Finally, we selected PSLY, PS (PSFP), PSLC, PC, and PK for metabonomics analysis. Potential markers were screened by partial least-squares discriminant analysis (PLS-DA) and S-plot analysis. It was found that the models of each group were good, and had a high explanatory and predictive degree for X and Y variables.

According to heat map analysis, adenosine, sucrose and pyroglutamic acid were the most suitable metabolites for identification the different *Polygonatum*; Sucrose, citric acid and pyroglutamic acid were the characteristic metabolites suitable for identification of *Polygonatum* germplasm from different regions. Adenosine, sucrose and pyroglutamate acid were the metabolites that could distinguish the above five kinds of *Polygonatum* samples. Correlation analysis was conducted to focus on the metabolites that distinguish the five *Polygonatum*, namely adenosine, sucrose and pyroglutamate acid. Adenosine was negatively correlated with sucrose and positively correlated with pyroglutamate acid. Sucrose has a significant negative correlation with adenosine and pyroglutamic acid. After that, through the metabolite box diagram analysis, it was found that adenosine, sucrose and pyroglutamic acid were the signature metabolites that could distinguish the different species and the different germplasm. Sucrose is the precursor of polysaccharide synthesis pathway, which provides a new direction for germplasm differentiation and related metabolic pathway research of *Polygonatum*.

Sucrose is the main product of photosynthesis and is widely distributed in plants. Sucrose is broken down by digestive juices in the body’s messaging system into fructose and glucose, which are absorbed through the small intestine (Szűts and Szabó-Révész, 2012). The sugars in sucrose are the building blocks of cells, such as their walls (Fewkes et al., 1971). Sugar is the main energy substance in living things. The sugars in sucrose are the precursors of many substances in the body, such as amino acids, nucleotides, fats, coenzymes, etc., all come from the intermediate products of glucose metabolism (Dissemond et al., 2020). Adenosine is an important intermediate for the synthesis of adenosine triphosphate (ATP), adenine, adenosine and adenosine (Novotný, 2015). Adenosine, as an endogenous nucleoside, can be phosphorylated to generate adenosine and participate in energy metabolism (O’Rangers, 1990). Pyroglutamate acid can stabilize intestinal structure, reduce intestinal inflammation and improve the structure and abundance of intestinal flora in mice fed a high-salt diet (Gunn et al., 2010). Furthermore, the development of plant metabonomics is bound to encounter a variety of difficulties and obstacles. Firstly, of prime importance is the limitation of the analytical methods (Kulkarni et al., 2018; Mai et al., 2020). Neither GC-MS or LC-MS can lengthy experiments (Fang and Luo, 2019). Secondly, data analysis methods are not efficient or convenient. Each metabonomics experiment generates a massive amount of data, and the task of data processing is heavy and complex (Li et al., 2015; Gargallo-Garriga et al., 2016). Consequently, more convenient data analysis methods are urgently needed to reduce the heavy workloads of researchers.

## Conclusion

This study provides a comprehensive profiling and putative identification of metabolites from *Polygonatum* through the UPLC-MS approach. For the first time, three different *Polygonatum* species were investigated simultaneously. After data screening and multivariate statistical analysis, the results revealed that the adenosine, sucrose, and pyroglutamic acid were suitable for identification of all five samples, which may be used for the further, more accurate identification of different *Polygonatum* species with potential as a chemical marker.

## Data Availability Statement

The original contributions presented in the study are included in the article/Supplementary Material, further inquiries can be directed to the corresponding author/s.

## Author Contributions

JN and SW designed the research. WL, XZ and GL performed the experiments and data analysis. SW and WL analyzed the data and wrote the manuscript. ZW and JN advised on the result and discussions. All authors discussed the results and implications and commented on the manuscript.

## Conflict of Interest

HC was employed by Shaanxi Buchang Pharmaceuticals Limited Company. The remaining authors declare that the research was conducted in the absence of any commercial or financial relationships that could be construed as a potential conflict of interest.

## Publisher’s Note

All claims expressed in this article are solely those of the authors and do not necessarily represent those of their affiliated organizations, or those of the publisher, the editors and the reviewers. Any product that may be evaluated in this article, or claim that may be made by its manufacturer, is not guaranteed or endorsed by the publisher.

## Footnotes

1
www.bioconductor.org

2
www.metaboanalyst.ca

3
http://www.hmdb.ca

4
http://metlin.scripps.edu/

5
http://www.massbank.jp/

6
http://www.lipidmaps.org

7
www.r-project.org

## Supplementary Material

Supplementary Table 1 List of the specific location data of different germplasms.

Supplementary Table 2 Raw LC/MS data of 30 test samples.

Supplementary Table 3 Potential biomarkers for different species of *Polygonatum* (PS/PC/PK/PSLY/PSLC).

Supplementary Table 4 Differences metabolites identified by MS/MS.

Supplementary Figure 1 Key constituents identified by MS/MS spectrometry.

Supplementary Figure 2 Z-score of differential metabolites.
