# Supplementary figures and images for: Metabolomics Study of Different Germplasm Resources for Three Polygonatum Species Using UPLC-Q-TOF-MS/MS
Source: Front Plant Sci. 2022 Mar 11;13:826902. doi: 10.3389/fpls.2022.826902 (PMC8963481; doi:10.3389/fpls.2022.826902)

S1 Figure The key constituents that identified by MS / MS spectrometry


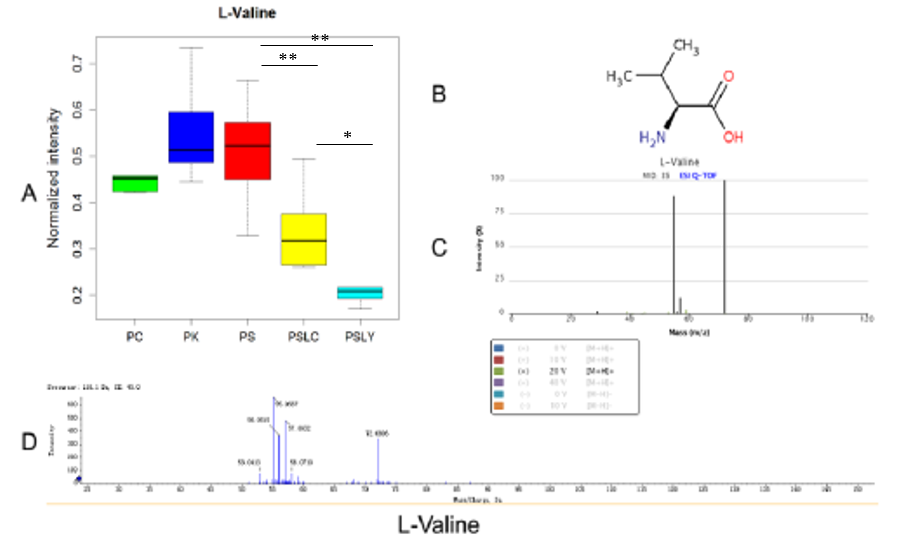

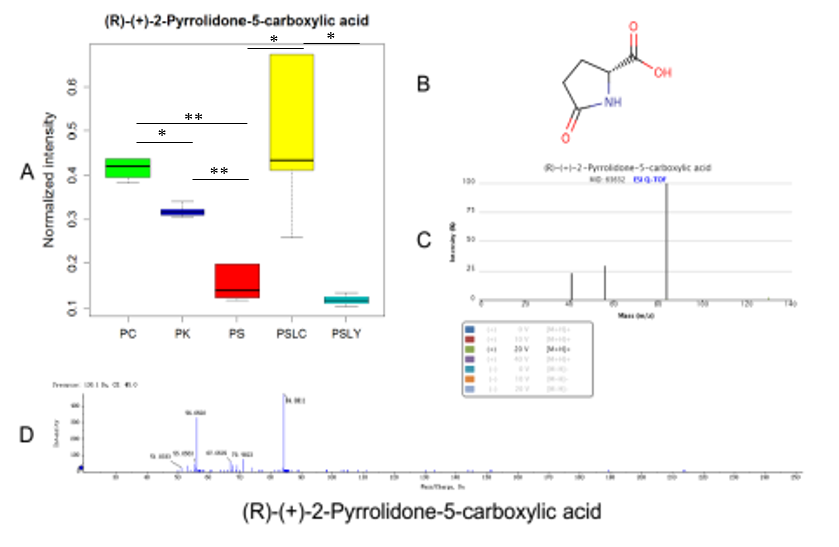


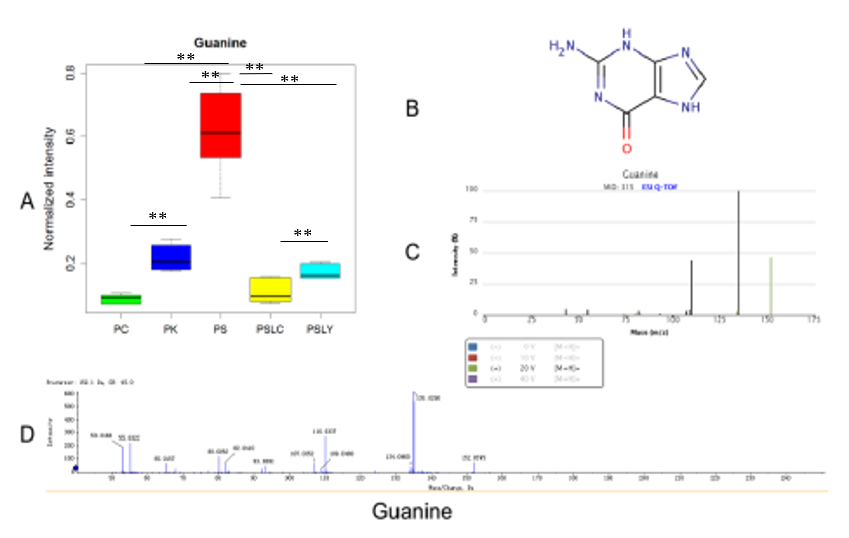

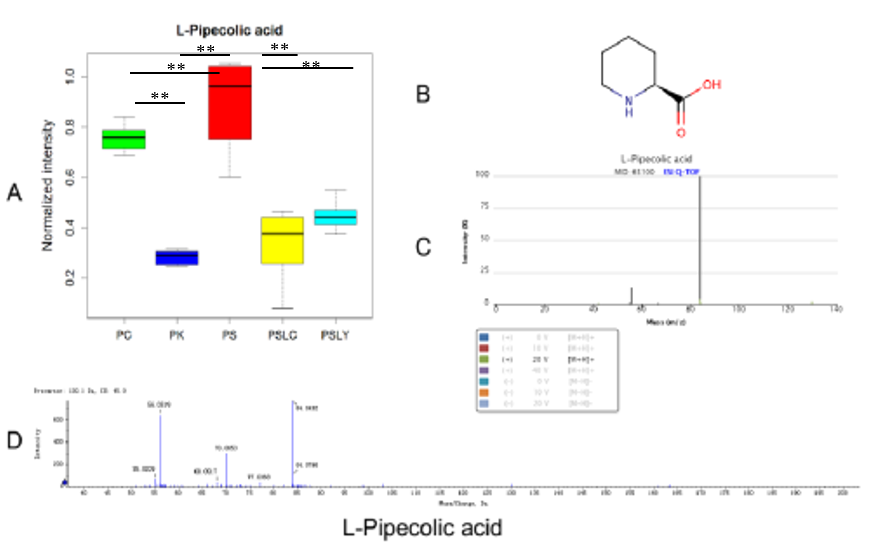


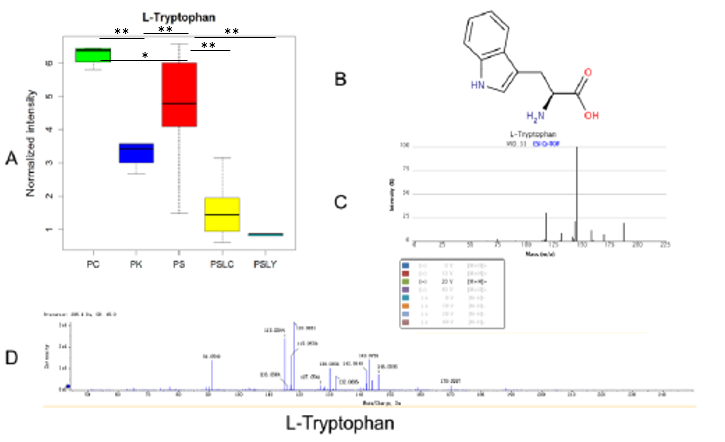

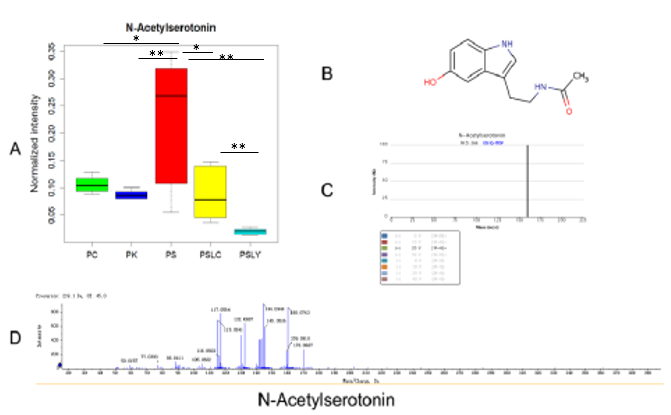


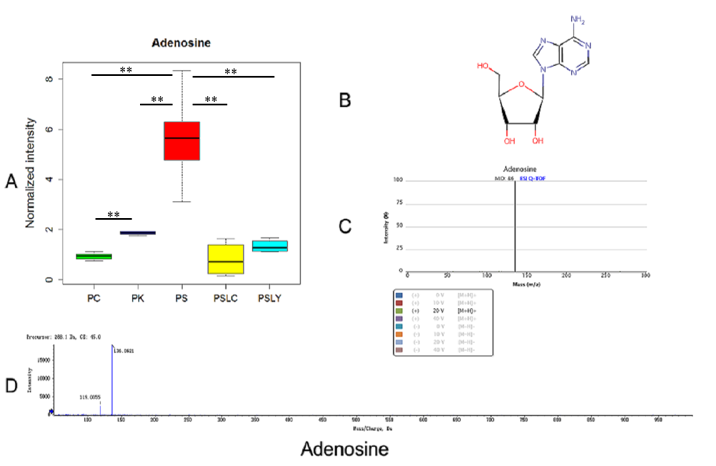

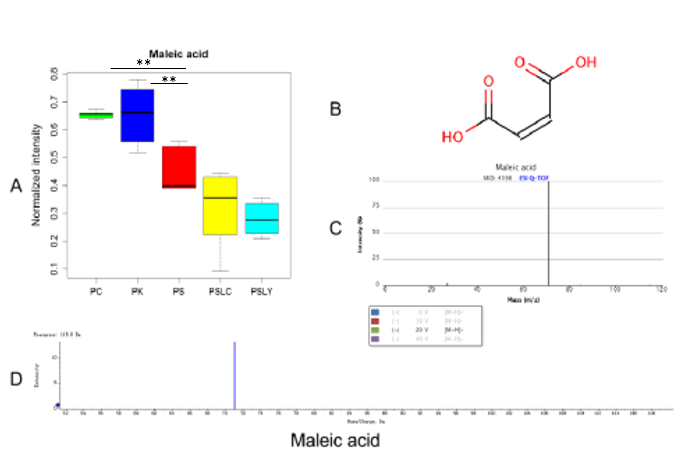


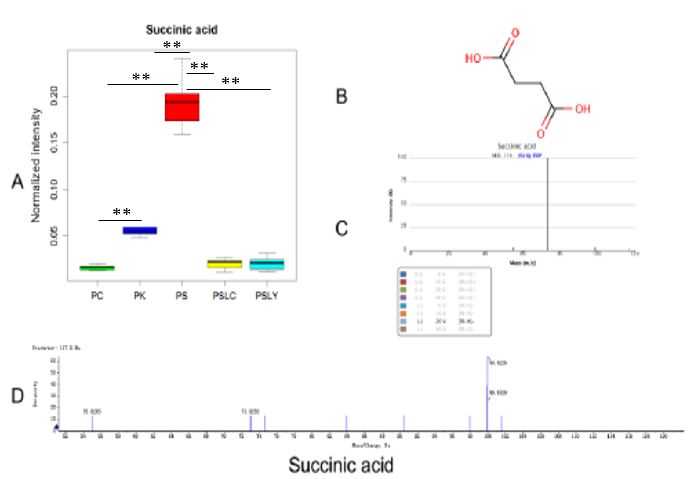

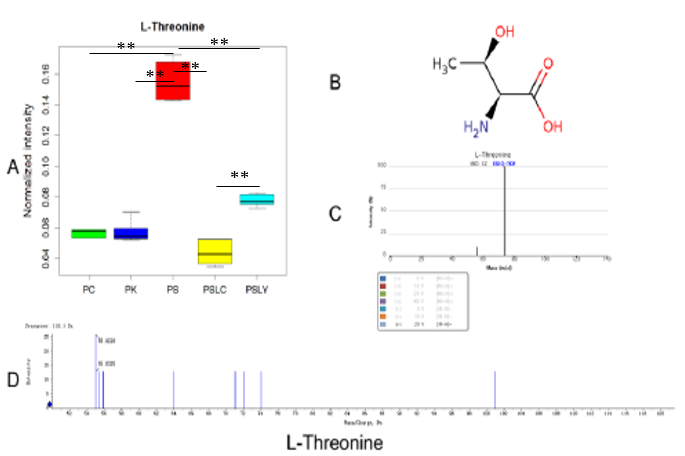


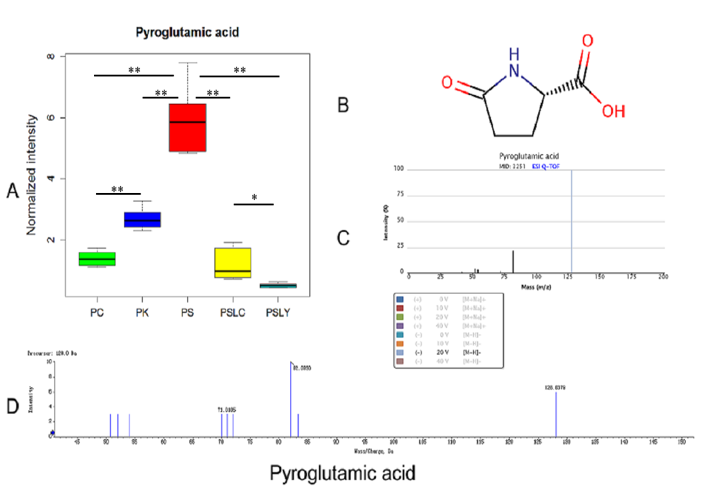

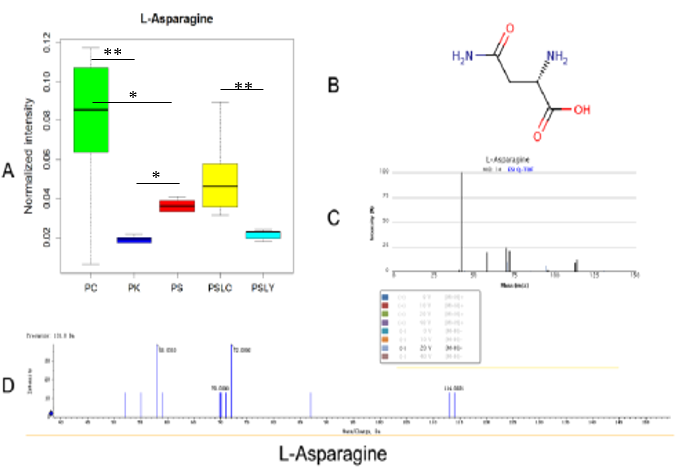


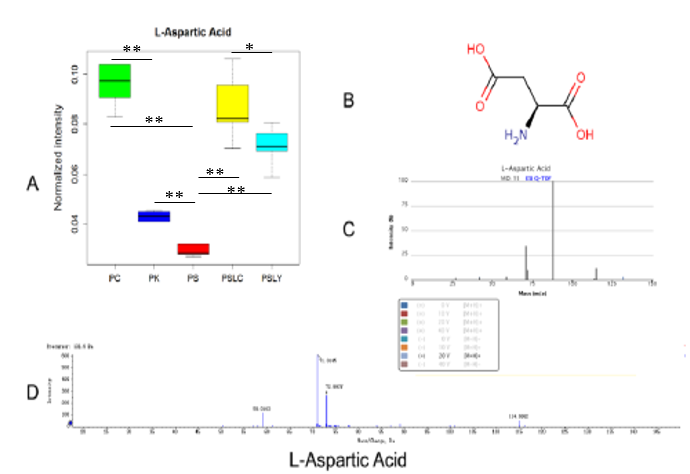

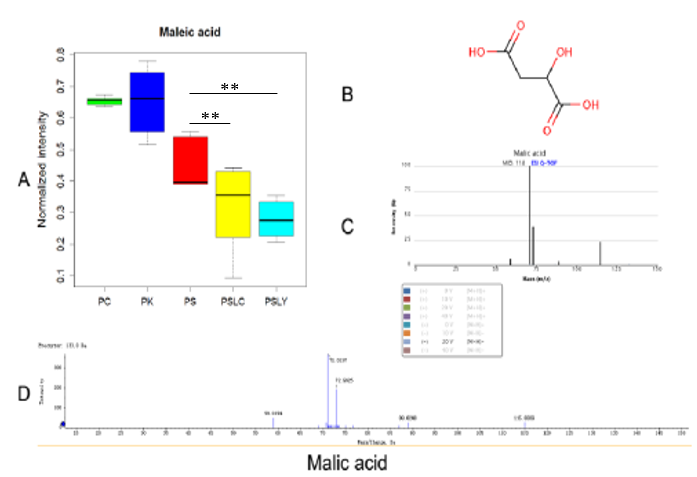


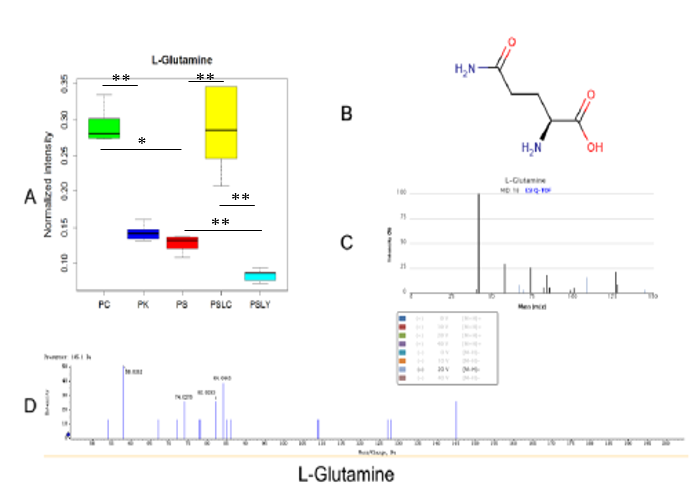

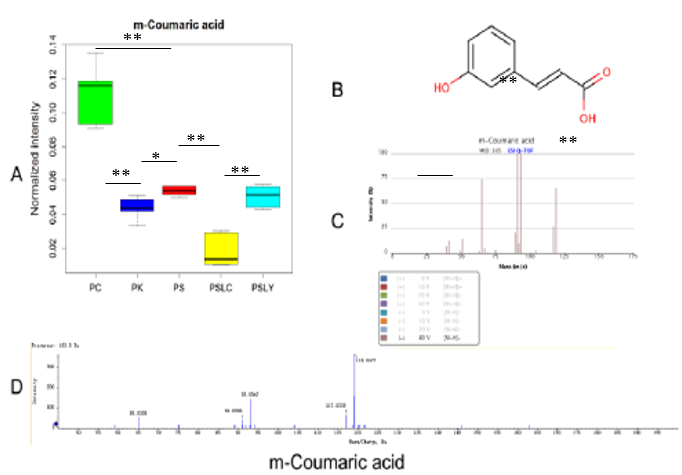


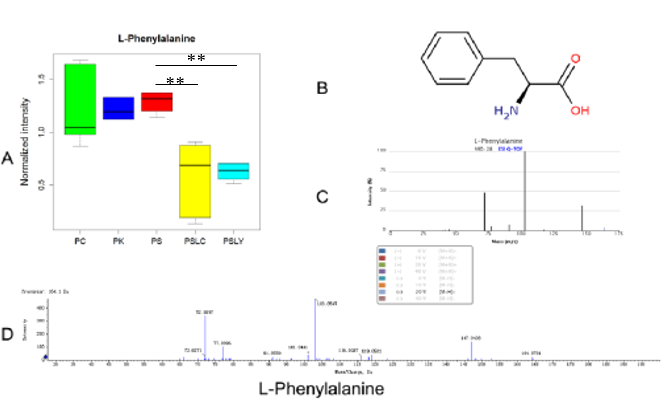

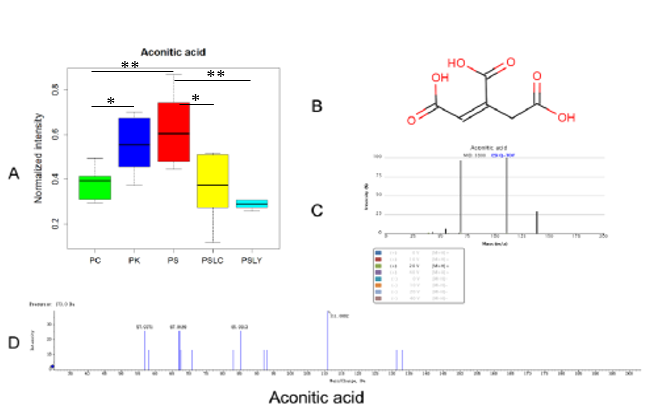


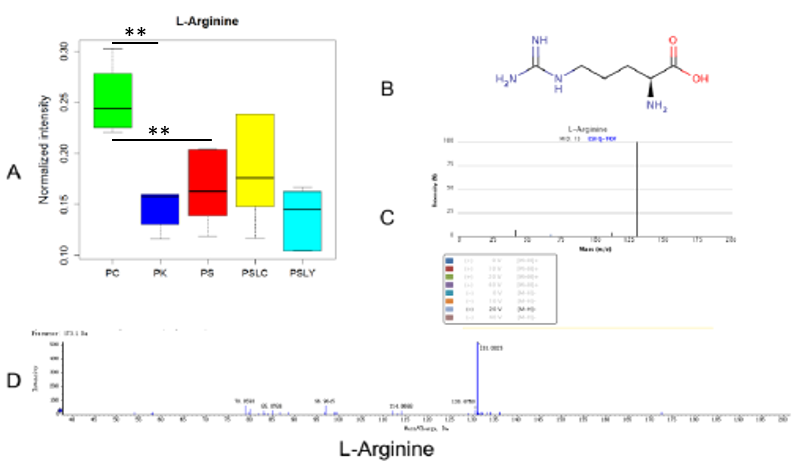

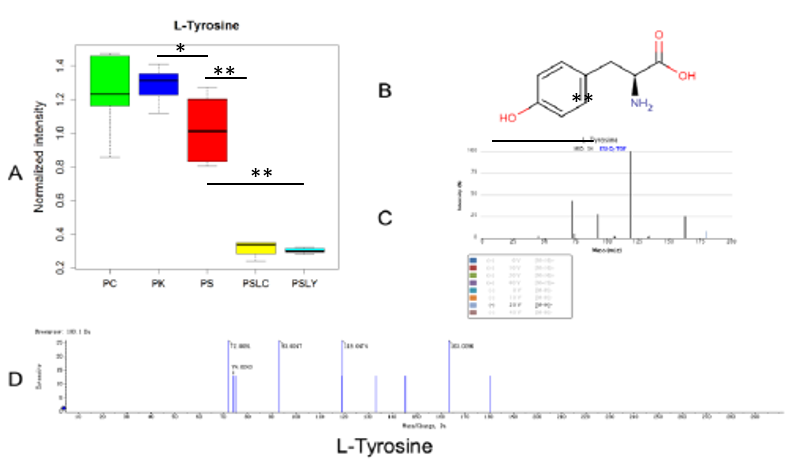


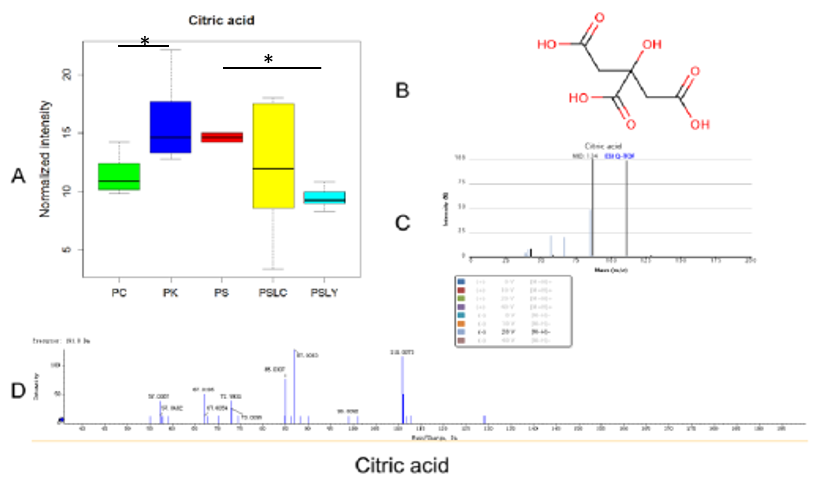

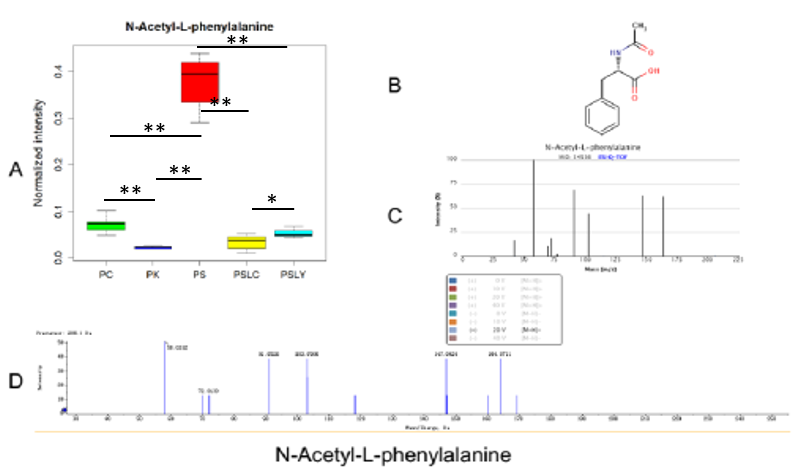


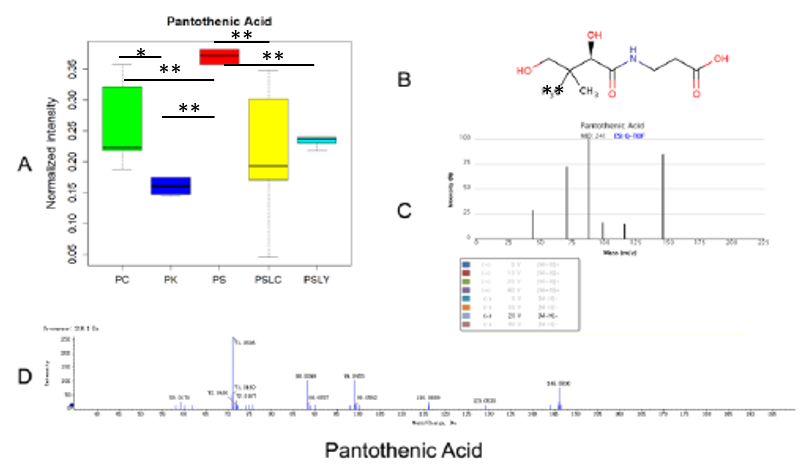

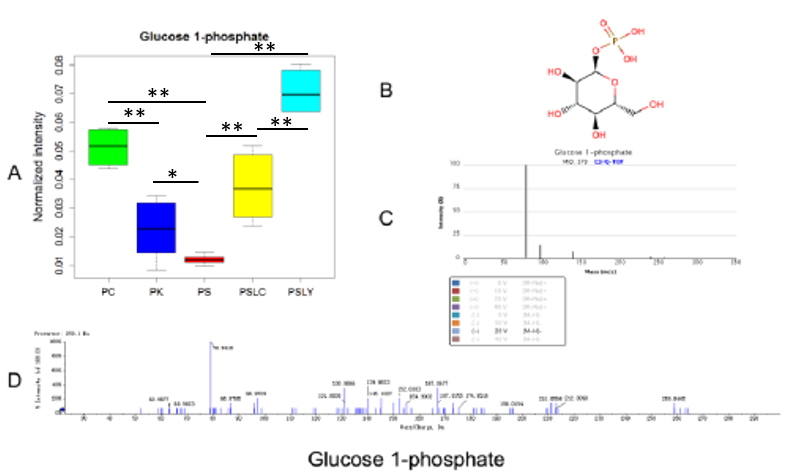


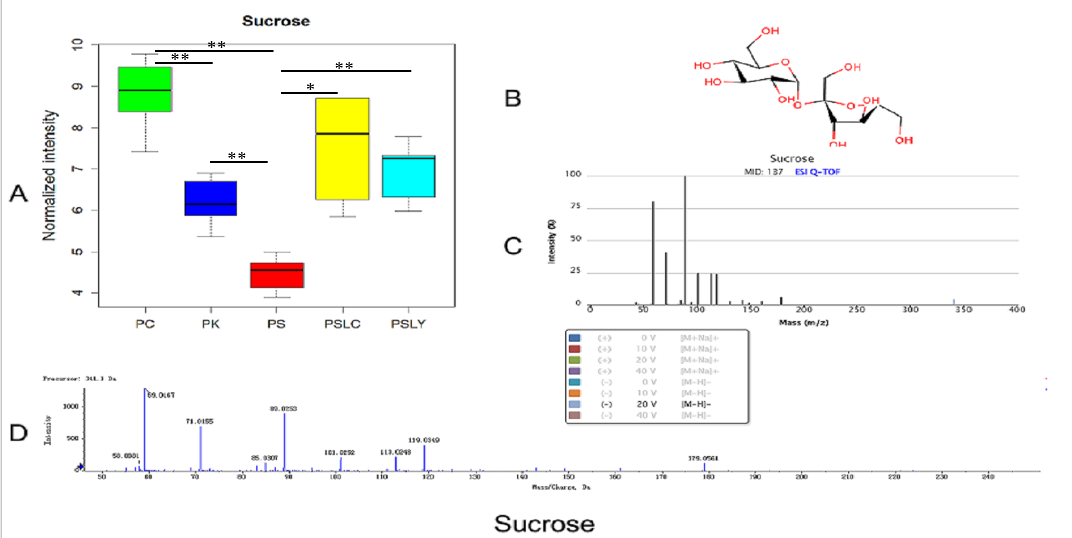

Supplement: Supplementary Table 2 — Raw LC/MS data of 30 test samples. [file Data_Sheet_1.ZIP › Supplementary Material/Supplementary Figure 1.docx]

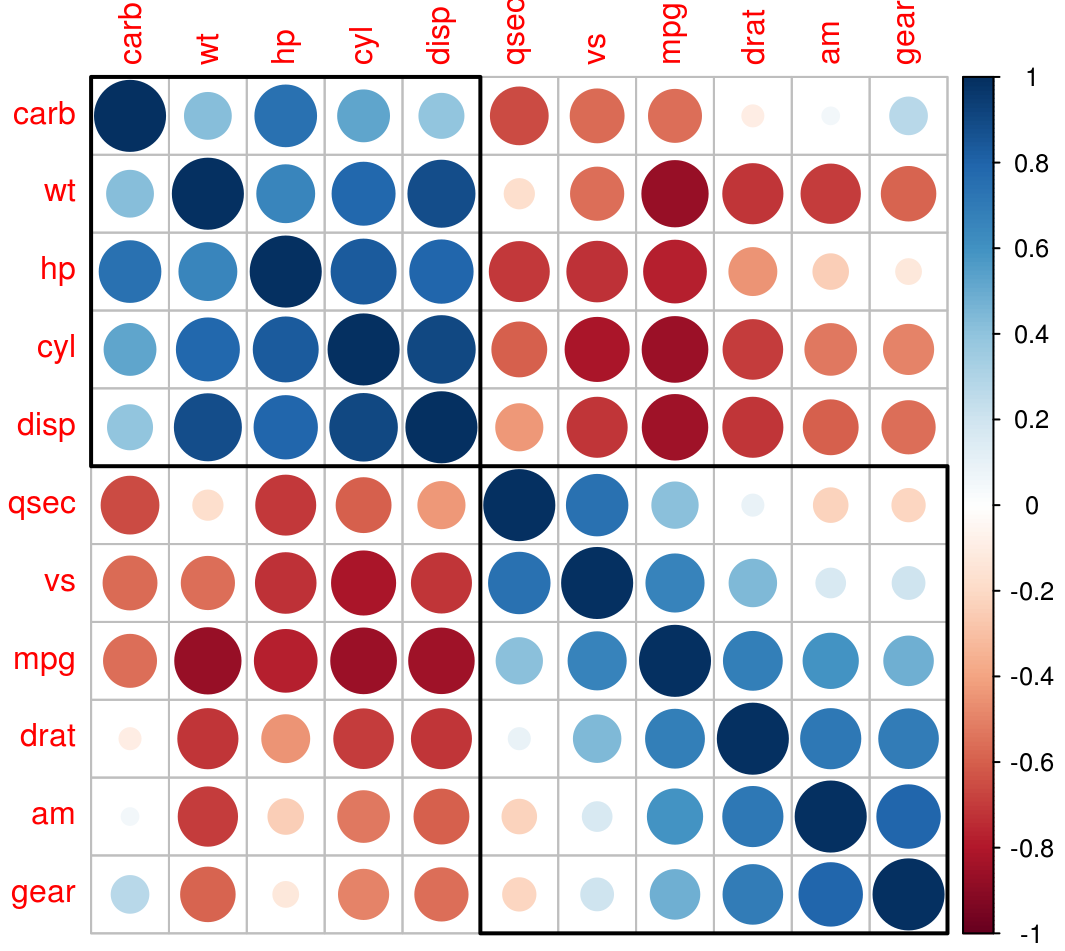

Supplement: Supplementary file 3 [file Data_Sheet_2.ZIP › corrplot-master/vignettes/webimg/rectangles-1.png]
